# Cultural considerations and beliefs surrounding preterm birth in Kenya and South Africa

**DOI:** 10.1186/s12978-023-01633-9

**Published:** 2023-06-12

**Authors:** Cecilia Milford, Emma Smith, Kenneth Ngure, Nicholas B. Thuo, Sara Newmann, Nalinie Lazarus, Mags Beksinska, Nelly Mugo, Larry Rand

**Affiliations:** 1 Wits MRU (MatCH Research Unit), Department of Obstetrics and Gynecology, Faculty of Health Sciences, University of the Witwatersrand, 11th Floor, Commercial City Building, 40 Dr AB Xuma Street, Durban, 4001 South Africa; 2 Albert Einstein School of Medicine, New York, USA; 3 School of Public Health, Jomo Kenyatta University of Agriculture and Technology, Nairobi, Kenya; 4 Center for Clinical Research, Kenya Medical Research Institute (KEMRI), Nairobi, Kenya; 5 Department of Obstetrics, Gynecology and Reproductive Sciences, University of California San Francisco, San Francisco, CA USA

**Keywords:** Preterm birth, Pregnancy, Culture, Beliefs, South Africa, Kenya, Intravaginal device

## Abstract

**Background:**

Preterm birth (PTB) is a global health epidemic, sub-Saharan Africa is severely impacted due to its limited healthcare resources. Pregnancy knowledge, cultural beliefs and practices play a role in the identification of risk and management of PTB. This study explored knowledge, understandings, cultural beliefs and attitudes to pregnancy and PTB, as well as cultural considerations for the introduction of an intravaginal device which could be used to identify PTB risk.

**Methods:**

Qualitative research was conducted in South Africa and Kenya. In-depth interviews were conducted using semi-structured guides with women with a history of PTB (n = 10), healthcare providers (n = 16) and health systems experts (n = 10); and 26 focus group discussions with pregnant women seeking antenatal care (n = 132) and community male partners/fathers (n = 54). Interviews/discussions were transcribed, translated, and analysed thematically.

**Results:**

Pregnancy knowledge, especially for first time pregnancies was poor, with many reporting late entry to antenatal care. Knowledge about PTB was understood in terms of gestational age, weight or small size of baby, with concerns about long term health and stigma. Various risk factors for PTB were described, including those related to traditions and beliefs of witchcraft/curses. Cultural practices, such as the use of traditional medicines and pica, and religion and its impact on health seeking behaviour were also viewed as risk factors. Although insertion of intravaginal devices was not widely acceptable in traditional communities, especially during pregnancy, it was felt that the use of one to detect risk of PTB would be accepted if proven effective in reducing PTB risk.

**Conclusions:**

Various culturally-informed beliefs exist which explain understandings of and attitudes toward pregnancy, pregnancy risk, and PTB. An inclusive exploratory process is critical to facilitate an understanding of the beliefs and traditions which could impact the introduction and design of a product to detect the risk of PTB.

## Background

Preterm birth (PTB), defined as delivery before 37 weeks of gestation [1–3], is a global health epidemic. More than 15 million premature births and one million associated neonatal deaths occur worldwide annually [2, 4]. In addition to being the global leading cause of mortality among children under five, those who survive are at significantly increased risk for long-term morbidity and lifelong disability [5–7].

The incidence of PTB is high in sub-Saharan Africa—a 2019 review noted that PTB rates in South Africa and Kenya were estimated between 100,000 and 249,999 births per country (10–14% and 5–9% of births respectively) [3]. PTB complications were ranked as the top cause of neonatal mortality in South Africa, and second in Kenya, in a review published in 2020 [8]. Teen pregnancy is a known risk factor for PTB and has been shown to have a high incidence in both countries [9, 10].

Although PTB has global significance, sub-Saharan Africa is especially impacted due to its lack of human resources, poor funding, and low resourced healthcare facilities [11]. PTB is associated with increased infant morbidity and mortality [3] with significantly high rates noted across Africa compared with developed countries [8]. Those preterm babies who survive are at increased risk for lifelong physical and psychological disabilities, which result in an economic burden to individuals and societies [5–7, 12].

Pregnancy knowledge, cultural beliefs and traditional practices may play a role in the identification of risk of PTB and management of preterm labour. Across Africa, there are strong beliefs in spirits or deities that affect perinatal outcomes [11]. For example, beliefs that obstetric complications could be due to bewitchment as a result of jealousy have been described in Mozambique [13], or due to evil spirits, disobedience, adultery or illicit sex have been described in Ghana and Nigeria [14–16]. Women from many African countries (including South Africa, Mozambique, Ghana, Sierra Leone, Cameroon, Zimbabwe, Nigeria, and Zambia) may hide their pregnancies and/or do not present early for pregnancy/birth care due to cultural beliefs and concerns of bewitchment which they believe could cause perinatal pains, harm to a foetus, and in some cases premature delivery [11, 13, 16–20]. Hidden pregnancies and inadequate prenatal care may also be risk factors for PTB. Beliefs about the causes of PTB may be both medical and traditional/religious—with participants from a study in South Africa citing bewitching, punishment by ancestors, and unfaithful partners as causes of PTB [21].

Culturally, different family and community members have different roles in pregnancies. For example, in Eswatini, mothers-in-law have authority over which health system beliefs and where and when pregnancy care is sought [11]. In some South African cultures, men/male partners do not play a role in pregnancy [22]. In other research, cultural beliefs are that traditional birth attendants/healers possess supernatural powers more capable to assist pregnant women, especially with complications, therefore women have failed to seek obstetric care [14].

Furthermore, vaginal practices and insertion of intravaginal products are influenced by culture and concepts of hygiene and sexuality [23, 24]. Vaginal practices and use of intravaginal products during pregnancy is not well documented.

A proof-of-concept device to identify women at high risk of preterm birth has been developed and is being tested in a clinical study for a high resource setting at the University of California San Francisco [25]. This novel intravaginal device measures microscopic changes in tissue density to identify in real time the structural changes in cervical collagen before symptomatic labour begins. Early detection of cervical changes may allow women more time to access care, which in turn could lead to improved pregnancy outcomes.

We conducted an exploratory, qualitative study on attitudes and preferences regarding an intravaginal diagnostic device, focusing on women with a previous history of PTB, healthcare providers, health systems experts, and other men and women in the local communities with pregnancy experience (attitudes towards, and descriptions of, a preferred device to detect risk of PTB are explored in more detail elsewhere [26]). This manuscript explores knowledge, understandings, cultural beliefs, and attitudes regarding pregnancy and PTB. In addition, cultural considerations for the introduction of an intravaginal device which can be used to identify risk of PTB are explored.

## Methodology

### Study population

This study was conducted in Kiambu County in Kenya, and KwaZulu-Natal province in South Africa, in late 2016 and early 2017. Representatives from five stakeholder groups, including women with a history of PTB, women currently pregnant and in prenatal care, male partners or fathers, healthcare providers, and health systems experts, were recruited via purposive or snowball sampling. The study details were presented to potential participants across groups, enabling an opt in approach for interested participants. In some cases, for example health systems experts, the potential participants provided referrals to more suitable/experienced experts.

Women with a history of PTB were recruited via community health workers, or word-of-mouth at healthcare facilities. Pregnant women attending antenatal services in urban or rural/peri-urban healthcare facilities were selected according to pregnancy status (> 20 weeks gestation) and age (16–45 years). Adult men (who had at least one child or a pregnant partner) were either partners of participating community females, or were recruited face-to-face through community male and youth groups. Community males and females, and women with a history of PTB had no prior relationship with interviewers.

Healthcare providers, including operational managers, doctors, nurses and community health workers, all with experience in maternity and/or antenatal services, were recruited face-to-face from urban or rural/peri-urban healthcare facilities. Health systems experts were selected and invited to participate based on their expertise in health policy, health economics, or regulatory issues, and many were found via word-of-mouth or referral. They were contacted telephonically or via email. Although in most cases there was no prior relationships with participants, where it did exist it was in the capacity of sharing professional expertise.

### Data collection

Data were collected by experienced social scientists and interviewers/research assistants from Kenya and South Africa, via in-depth interviews (IDIs) with health systems experts, healthcare providers and women with a history of PTB; and focus group discussions (FGDs) with community men and pregnant women. All interviewers/facilitators had training and experience in qualitative research and the specific study requirements. Interviewers/facilitators were matched with participant type. For example, female interviewers/research assistants conducted IDIs/FGDs with community females/pregnant women, and male interviewers/research assistants interviewed community men. More experienced (including masters/PhD level) researchers conducted the interviews with some of the healthcare providers and the health systems experts. A similarly matched notetaker was present, in addition to the facilitator, and took detailed notes during the focus group discussions.

IDIs and FGDs with PTB women and community men and women were held in community halls, healthcare facilities, and/or venues convenient to the participants. IDIs with healthcare providers were held at healthcare facilities, and IDIs with health systems experts at a venue of their choice, such as their offices, facilitating convenience and comfort for participants. Sufficient IDIs and FGDs were planned and conducted for data saturation to be reached.

Semi-structured interview guides were used, and interviews and discussions lasted approximately 1–1.5 h. IDIs and FGDs were conducted in English or *isiZulu* (South Africa) or in English or *Kiswahili* (Kenya), and were audio recorded with permission from the participants. During the discussions, participants were shown two examples of intravaginal devices available in some markets (but not widely used in Kenya or South Africa)—the FemCAP and a pessary—to guide discussions about acceptability of an intravaginal diagnostic device. This was part of a larger study to explore preferences and inform the design factor for an intravaginal diagnostic devise to detect risk for preterm birth [26]. In addition to exploring views and attitudes towards an intravaginal diagnostic device, participants were questioned and prompted about community definitions of pregnancy and their community’s thoughts on causes of PTB. Although a variety of medical and non-medical reasons were described as risk factors for PTB, this manuscript focuses on traditional and cultural perspectives of pregnancy and PTB, which may inform the introduction of an intravaginal diagnostic device.

### Data coding and analysis

IDIs and FGDs were transcribed and translated directly from *Swahili *and/or *isiZulu* (where necessary) into English. Transcribers of the non-English interviews/discussions were native *Swahili* or *isiZulu* language speakers who were also fluent in English. They were research assistants, data collectors and/or transcriptionists who were trained in the study with a background in sexual and reproductive health research. They listened to the audios and transcribed these directly into English.

Dedoose (https://www.dedoose.com/), a qualitative data analysis software program, facilitated organization and coding of data. Thematic analysis was used to explore knowledge, understandings, cultural beliefs, and attitudes regarding pregnancy and PTB. In addition, cultural considerations for the introduction of an intravaginal device to identify risk of PTB were explored.

Two researchers from each country (CM, NL, NBT and a research assistant) independently reviewed three to four transcripts each (representing FGD and IDI data from across both countries), and drafted a code list per country team. These country code lists were merged to create a single draft code list with definitions of codes, which was relevant, applicable, reliable and valid, across both countries. Codes were generated iteratively based on input from the questions in the guides as well as from emergent themes. Themes were identified by reading the transcripts and identifying repetitions, categorizations, similarities and differences, and from theoretical interpretations of the data [27]. The initial draft code list was tested by double coding across countries—a portion of transcripts (n = 9) were double coded and inter-coder comparison discussions were conducted to determine overlap in coding and understandings of code names and definitions. This was done to facilitate reliability and validity of coding across countries. Based on these discussions, new codes were identified and differences in coding were discussed, until agreement in definitions were reached. This process was performed multiple times until there was full agreement. The master code list was updated once there was agreement based on these discussions.

Data analysis was conducted as a team effort across Kenya and South Africa. Each country had their own teams (two coders per country) who coded their own transcripts (a portion of which were also double coded at a country level). There was ongoing communication about coding, and any coding queries were collaboratively resolved throughout data analysis. This process facilitated reliability and validity of coding between and within the two countries.

### Ethical considerations

Voluntary, written informed consent was provided by all participants. For those participants who were under 18 years of age, written parental consent and individual written assent were obtained. Ethical approval was obtained from the Kenya Medical Research Institute-Scientific Ethics Research Unit (KEMRI-SERU) in Kenya (KEMRI/SERU/CCR/0028/3240), and the Human Research Ethics Committee (HREC) of the University of Witwatersrand in South Africa (M160131). Additional support and approval were obtained from the Kiambu County Health Research and Development Unit and participating healthcare facilities in Kenya, and from the Department of Health (Provincial and District), and participating healthcare facilities in South Africa. Approval was also granted by the University of California San Francisco Institutional Review Board.

## Results

The majority of participants were female (n = 87 and n = 55 in Kenya and South Africa respectively), and most were between the ages of 20–24 years. The mean age of male participants was higher, at 31 and 30 years in Kenya and South Africa respectively. Healthcare providers were predominantly female in both countries. There were more male health systems experts in Kenya. Details of study participants are outlined in Table 1.

**Table 1:** Demographic details of participants

Participants	Kenya n (%)	South Africa n (%)
Female participants:	87 (100)	55 (100)
In-depth interviews	5 (6)	5 (9)
Focus group discussions	82 (94)	50 (91)
16–19 years	25	12
20–24 years	29	25
25–45 years	24	13
Parity, mean (Range)	1 (0–5)	1 (0–3)
History of PTB	8	8
Male participants (FGDs):	17 (100)	36 (100)
Age, mean years (Range)	31 (24–40)	30 (21–42)
Parity, mean (Range)	2 (0–5)	2 (0–7)
History of PTB	1	9
Healthcare providers:	8 (100)	8 (100)
Female	5 (63)	7 (88)
Age, mean years (Range)	41 (29–55)	44 (27–61)
Health systems experts:	5 (100)	5 (100)
Female	2 (40)	3 (60)
Age, mean years (Range)	42 (31–53)	52 (40–66)

Thematic areas first highlight participant’s knowledge and understandings of pregnancy, including pregnancy health seeking behaviour, and knowledge and attitudes towards preterm birth. Following this, beliefs, cultures and traditions surrounding the causes of preterm birth are described. Finally, attitudes towards the use of intravaginal devices during pregnancy are explored. When there is agreement among both countries according to the thematic area discussed, illustrative quotes from either country are provided; and where there are inter-country differences, quotes from both countries are included. Perspectives from different stakeholders are presented in an integrated manner, and differences in opinions are highlighted.

### Pregnancy knowledge, understandings and health seeking behaviour

In South Africa, and Kenya it was felt that people do not know a lot about pregnancy, especially with first time pregnancies.

*There is ignorance and lack of information when the woman is pregnant. [Kenya, rural, adult males, FGD]*

*For those that are […] the primigravidas sometimes they don’t know what is labour. […] That are pregnant for the first time, […] because we they are just fantasizing because if I’m, and it depends on the information that she was given, if ever there was a evidence that she was taught. [South Africa, healthcare provider]*

However, in both countries, participants from all groups demonstrated an understanding of the duration of a normal pregnancy. They described that it lasts 38–40 weeks (or 9 months), and some suggested it could be less—at 36 or 37 weeks. A few healthcare providers suggested that normal pregnancy may go on to 42 weeks.

#### Health seeking behaviour

Although healthcare providers recommended that pregnant clients should start attending antenatal clinic (ANC) visits before 20 weeks, in both countries many participants reported that entry to care was variable with late or inadequate prenatal care.

*[Some women] start their ANC early, […] at one month and others depends, at seven months. [South Africa, healthcare provider]*

### Preterm birth: knowledge and attitudes

South African and Kenyan respondents understood preterm birth in terms of gestational age, low weight or small size of baby, or whether delivery occurred before nine months of age.

*If you give birth before time, that means the months are not yet nine. […] The time has not yet come, that you can give birth to that baby. [South Africa, urban, adult female, FGD]*

***Interviewer (I): What would make you think she delivered before nine months?***

*Participant (P): Their [baby’s] body size, you know you look if the body size is small. [Kenya, woman with a history of preterm birth, IDI]*

#### Attitudes towards preterm babies

In South Africa there were no major discussions about concerns with preterm births per se. Rather, discussions indicated that it was preferable for people to have healthy, “big” babies.

*[P]eople don’t want preterm babies, they don’t want small babies. Every woman wants to tell you “I wanna big baby”. [South Africa, health systems expert]*

In both countries, there were concerns about the long term health implications and possible death of a preterm baby. In addition, concerns of stigma of preterm babies were described.

*[Y]ou will find that the child who was born prematurely, maybe at birth there were body parts which were not fully developed and that child will live in and out of hospital being sick and you will find that in… in the community you usually hear that child is retarded because they were born prematurely, they were put in the nursery and such. [Kenya, urban, young adult female, FGD]*

### Beliefs, cultures and traditions surrounding causes of preterm birth

Respondents understood various risk factors for preterm birth, including (young or old) maternal age, physical activity, unhealthy diet, medical conditions such as hypertension, and previous preterm birth. They also described various risk factors related to beliefs, cultures and traditions.

#### Witchcraft

Community members from both South Africa (largely rural male and female members) and Kenya described the belief that PTB could be caused by witchcraft (*umeqo* in South Africa, *uchawi* in Kenya*)*.

*I think that sometimes it’s things that people believe in, […], that traditional things, those who believe in isiZulu traditional things, they believe that you have been bewitched and that is why the baby got affected, and it inhaled bad spirits, all that, you see. Those are things that people believe that one had preterm birth. [South Africa, rural, adult female, FGD]*

*[T]here are those I usually hear them say that according to culture you might be bewitched or it might be a familial thing in your family that you get premature babies. [Kenya, rural, female teen, FGD]*

Witchcraft was believed to be used when interpersonal relationships were strained. Participants from both countries described that negative relationships with male partners could result in witchcraft and a preterm birth, for example when there were marital disagreements.

*P: In my community they strongly believe in traditional medicines, so if you had miscarriage they just say you were bewitched they believe that the woman you are sharing a man with bewitched you, they believe in such things.*

***Facilitator (F): But if you didn’t have miscarriage but you had preterm birth, what do they say?***

*P: It also happens like that and by your luck baby survives you see, and the cause was that, but you find that your baby gets saved and be able to be born alive. [South Africa, rural, female young adult, FGD]*

In addition, Kenyan participants described other relationship issues, such as jealousy from others and curses from parents/grandparents, that could cause PTB.

*I usually hear some people in the community say that if you meet someone on the way and you are pregnant and perhaps they don’t have a baby and they are jealous, they can bewitch you and make you get a baby prematurely. [Kenya, rural, female teen, FGD]*

*Others say that, maybe a woman is married somewhere she is not wanted by her in-laws; you see the curses from the parents can cause the child to be born before term. [Kenya, rural, adult female, FGD]*

#### Traditional medicine

In South Africa, community members and healthcare providers described beliefs that using traditional medicine or African herbal health tonics could cause preterm birth.

*Some people believe that if you drinking “isihlambezo” [a herbal health tonic] it causes a baby to come too fast. [South Africa, woman with a history of PTB, IDI]*

#### Pregnancy cravings

South African participants also described that common pregnancy cravings, such as a non-nutritive pica (eating soil during pregnancy), could lead to preterm birth.

*P: Maybe sometimes it depends on things that you eat. Sometimes you eat wrong things like ‘ukhetho’ [a kind of soil that people eat—as a craving during pregnancy], they say, it makes you to have miscarriage before time. It depends on things that you eat sometimes.*

***F: Okay what is ‘ukhetho’?***

*P: It is the soil that people eat ‘umcako’ [white wash—also eaten during pregnancy, a craving]. […] They say it causes a damage in the bladder and it makes the uterus get opened early and give birth prematurely. Maybe 7 to 8 [months] or so or have a miscarriage straight. [South Africa, rural, adult female, FGD]*

#### Religion and fate

Some female community members (from the rural area) in South Africa attributed preterm birth risk to fate—with spiritual or religious connotations:

*I think that sometimes it can be spontaneous because it is God who makes the baby. So sometimes, it can be spontaneous without any certain cause. It can be spontaneous that it is born before time. [South Africa, rural, young adult, female, FGD]*

In Kenya, religious beliefs were linked with health seeking behaviour, which was described as a factor which could cause preterm birth. More specifically, some religious denominations in Kenya have beliefs and taboos surrounding vaginal examinations and fears of HIV testing, therefore pregnant women may choose to have community support during delivery and avoid ANC visits, putting them at risk for PTB. Similarly, South African participants described that they traditionally only seek help when in pain, and therefore may not attend ANC visits early in pregnancy, also putting them at risk for PTB.

*There is a certain religion who usually dress in long dresses […] they rarely come for the maternity services, they only come with a baby for BCG [vaccination: Bacillus Calmette–Guérin] and when you ask about them, “I gave birth at home assisted by a church member”. [Kenya, healthcare provider]*

*[T]he issues of the culture as I have already said it- […] that they don’t usually come to our clinics very early we are struggling a lot… in terms of early ANC booking at 20 weeks- […] You understand that you are already 20 weeks but we are very low in terms of the numbers because patients they come to, they want to come they are always regarding themselves as normal people [not pregnant] […] So they don’t have to come early. [South Africa, health systems expert]*

*Because…with us [referring to black South African women], [laughs] I don’t know with other cultures, […] as African, we only seek help when there is pain. [South Africa, healthcare provider]*

#### Termination of pregnancy

In both South Africa and Kenya, preterm birth was believed to be related to termination of pregnancy (TOP), with many viewing PTB as a failed termination.

*I used to hear that some other people, especially those who gave birth at 7 months saying that she was trying to do abortion or something. […] They used to say that maybe she was trying to abort the baby that is why she gave birth prematurely. [South Africa, urban, young adult, female, FGD]*

In Kenya, participants also described beliefs that women who have had a previous TOP may have a PTB in future.

*If you usually have induced abortion at 5 months of pregnancy, there is a possibility of the body expelling subsequent pregnancy at 5 months of pregnancy because the body is used to. [Kenya, woman with a history of PTB, IDI]*

*[L]et’s say someone has… a woman is pregnant and maybe has a tendency of inducing abortions in the past, that can also cause a child to be born too early. Let’s say she gets pregnant, it can cause a woman to give birth to a child before 37 weeks if they have a tendency of inducing abortion. [Kenya, urban, young adult female, FGD]*

### Attitudes towards intravaginal devices during pregnancy

There were various opinions about the insertion of products into the vagina in general. Some respondents felt that women may be uncomfortable with self-insertion practices and that culturally they don’t like to touch their vaginal areas.

*You see again in African setup you know if there’s anything going inside [the vagina] … that system is not very welcome. [South Africa, health systems expert]*

*They do not like touching their genitals, they even do not know how they look like; so I am wondering whether they will accept to wear these things [intravaginal devices].[…] Even there are some you want to do a vaginal examination they refuse and you maybe want to know the dilatation of the cervix, she refuses; she is normally not touched there [vagina], so they take time to…to make them accept. [Kenya, healthcare provider]*

More specifically, health systems experts in South Africa and providers in Kenya spoke about the unacceptability of inserting anything into the vagina during pregnancy, both in the medical fraternity and in the community.

*[T]here is a baby and there are a lot of myths and taboos about putting intrusive things and how the baby will actually come out as. I would give you a typical example because I am deriving it from other experiences. When you have an intrauterine device and a woman conceives with it, they believe the baby will come with it stuck here [touching her head] or it has gone in the baby’s brain. [Kenya, health systems expert]*

Community members from both South Africa and Kenya had concerns that touching the vagina could be unhygienic and even harmful to unborn babies.

*I think other people will be afraid that they will be having this thing [an intravaginal device] inserted into their womb during their pregnancy, [I: Cervix.] on the cervix, because they will be afraid that if it happens that it harms the baby. [South Africa, woman with a history of PTB, IDI]*

Insertion of a device during pregnancy was described as forbidden in some African cultures. Some community males from Kenya expressed concern that the use of an intravaginal device during pregnancy could be viewed as witchcraft.

*P4: She might think it is witchcraft, she might think you are monitoring her…that thing of eeeh it is hard. […] Because she might think that you have visited a witchdoctor (name of a witchdoctor) and you are inserting it to monitor her so she doesn’t…*

*P5: So that she doesn’t abort [Kenya, urban, adult male, FGD]*

However, in spite of traditional beliefs, respondents from both countries (including community males and females as well as healthcare providers and health systems experts) felt that if an intravaginal product was perceived as beneficial, safe, and they were appropriately trained on it, it would largely be acceptable, and community women may use it.

***F: So do you think women in your community like in [place in Kenya] would want to use a device like this? […]***

*P8: If they are told of the importance and they are trained about that device some can accept. […] Again they will have to ask if this device has been used elsewhere. If the answer is no (chuckles) there will be so many questions on the same. […]*

*P7: Now the problem here in Africa is if they hear it is not used in any other place but if they hear there is a country where that device is being used and it is helping them I don’t think they will also reject that device. They will be very happy as long as it doesn’t have side effects. [Kenya, rural, adult male, FGD]*

*It’s my experience that intravaginal devices have not been very successful, especially in Africa. And it may be due to the myth that the woman is using a a device. If you look at eh, for example the use of a female condom. The female condom has not taken off in Africa and I think it’s a probably lack of education, lack of understanding, lack of know-how on how to use it. […] But you must always remember that the African patient is very receptive. And if she understands that whatever you’re doing for her is going to reduce the risk of her giving birth to a preterm baby which is, babies are very precious in Africa, you know… […] No woman wants to not to have a baby. [South Africa, health systems expert]*

However, community females from South Africa felt that more traditional women may be less likely to use an intravaginal preterm birth detection device than young, open minded women.

*P6: I can say that there can be a group of people who would like to use it and a group of people who wouldn’t like to use it in the community, and in my community, because a lot of people have grown up and they are women like this and they mostly believe in traditional things. So, they can have a lot of questions like “what if this thing hurts the baby or it gets lost while inserted?” and stuff. And then maybe there is youth like this, who have knowledge who would like to use the device. […]*

*P2: I agree with what number 6 says. Most of the time youth will be able to use it and adults will not agree to use it. [South Africa, urban, female teen, FGD]*

## Discussion

In this study, similar to other research in South Africa [22], providers and male community members felt that pregnancy knowledge was poor, especially among adolescents and primigravidas. Despite this, participants demonstrated understandings of the duration of normal pregnancies. Participants had discussions about PTB and what this meant to them, and this was described in terms of gestational period, size and weight of the baby.

Reports of ANC attendance in this study were varied -with some women attending early and others late in pregnancy. Reasons for late attendance may be linked to multiple factors, including parity and unplanned pregnancies. Previous research has also demonstrated that cultural beliefs or concerns of bewitchment could lead to late ANC attendance [11, 13, 16–20]. Late ANC, or fewer ANC visits could also be a risk factor for PTB [28].

Many beliefs exist about the causes of and risks for PTB. Respondents in this study described physical risk factors also noted elsewhere, such as maternal age [28–30], unhealthy diet, medical conditions (such as hypertension) [28] and previous history of PTB [31]. Previous or attempted termination of pregnancy was also viewed as a risk factor for PTB, although contemporary methods for pregnancy termination are not associated with increased risk for PTB [32].

Furthermore, some understandings of PTB are rooted in beliefs around witchcraft and other spirits or deities that could affect pregnancy and birth, as has been found in other studies across Africa [11, 13–16]. Specifically, witchcraft has been described as playing a role in PTB when interpersonal relationships were not good, either between couples (for example jealousy, adultery, etc.) [13–16, 21] or in cases where pregnant women do not take advice from authoritarian figures in families (e.g. mothers-in-law) [11]. Similarly, spiritual, supernatural and social beliefs—witchcraft and “god’s plan” have also been found to explain pregnancy losses/stillbirth in a study in Kenya and Uganda [33].

Cultural practices, such as the use of traditional tonics and pica (eating soil), were also believed to put pregnant women at risk of PTB. Such beliefs and attitudes could result in stigma around PTB. Furthermore, poor birth outcomes and long-term health complications [5–7] in individual babies born before time, were viewed as potentially stigmatising by participants in this study. Community members reportedly had a relatively negative perspective about premature babies.

The use of an intravaginal device to identify those at risk of preterm labour could potentially allay morbidity of PTB. However, acceptability of use of an intravaginal device is also closely linked to culture and traditions. In general, participants described that vaginal practices, vaginal touching, and insertion of intravaginal products were not widely acceptable in more traditional communities. More specifically, insertion of products intravaginally during pregnancy was described by participants in this study as unacceptable and even forbidden in some cultures, and there is a paucity of other research documenting this. Despite concerns about inserting a product intravaginally during pregnancy, community members and healthcare providers felt that pregnant women may be willing to wear an intravaginal diagnostic device, if it was shown to be safe and effective in reducing the risk of PTB.

As with any study there are some limitations. Qualitative data are limited in generalizability, and the data from this study are specific to regions in two Sub-Saharan African countries. However, the data from this study are exploratory, and highlight traditional and cultural considerations which could impact on PTB experiences and health seeking behaviour.

## Conclusions

Various culturally-informed beliefs exist which explain understandings of and attitudes toward pregnancy, pregnancy risk, and PTB. These beliefs may result in stigma and may perpetuate myths about pregnancy risk. In this study, before the implementation of an intravaginal device to detect possible PTB risk, a comprehensive consultative process was undertaken with male and female community members, healthcare providers and stakeholders, to explore beliefs, attitudes, and understandings. An inclusive exploratory process is critical to facilitate an understanding of the beliefs and traditions which could impact on the introduction of a product to prevent preterm birth. Taking cognizance of the interaction of cultural beliefs and modern medicine is necessary to inform end-product design.

## Data Availability

The datasets used and/or analysed during the current study are available from the corresponding author on reasonable request.

## Abbreviations

ANC: Antenatal clinic
HREC: Human Research Ethics Committee
F: Facilitator
FGD: Focus group discussion
I: Interviewer
IDI: In-depth interviews
KEMRI-SERU: Kenya Medical Research Institute- Scientific Ethics Research Unit
P: Participant
PTB: Preterm birth
TOP: Termination of pregnancy
